# Efficacy and safety of biologic agents for IgA nephropathy: A protocol for systematic review and meta-analysis

**DOI:** 10.1371/journal.pone.0298732

**Published:** 2024-03-28

**Authors:** Jia Ma, Jianyue Xing, Yupeng Zhang, Guangzhen Liu

**Affiliations:** 1 Shanxi Traditional Chinese Medical Hospital, Taiyuan, China; 2 Shanxi University of Chinese Medicine, Taiyuan, China; University of KwaZulu-Natal, SOUTH AFRICA

## Abstract

**Background:**

IgA nephropathy (IgAN) is the most prevalent primary glomerulonephritis worldwide and a leading cause of chronic kidney failure. There are currently no definitive therapeutic regimens to treat or prevent the progression of IgAN. However, biologic agents offer novel therapeutic approaches that target immunological mechanisms to slow or halt disease progression. The objective of this study is to evaluate the efficacy and safety of biologic agents in patients with IgA nephropathy.

**Methods:**

We will systematically search PubMed, EMbase, Web of Science, Cochrane Library, and www.clinicaltrials.gov for randomized controlled trials of biologic agents for the treatment of IgA nephropathy. The search period will span from the establishment of each database until October 2023. The quality assessment of included studies will be performed individually using the revised Cochrane risk-of-bias tool for randomized trials (RoB 2), and meta-analysis will be conducted using Revman 5.4.1 software.

**Conclusions:**

The results of this study will provide evidence-based medical evidence for the clinical application of biologic agents in patients with IgA nephropathy.

**Prospero registration number:**

CRD42023400450.

## Introduction

IgA nephropathy (IgAN) is the most prevalent primary glomerulonephritis worldwide, characterized by the deposition of immunoglobulin A (IgA) in the glomerular mesangium [1, 2]. IgAN is more common in Asians than in white populations but less common in black race, and it is generally more common in males than in females in the gender distribution [3–5]. The main clinical manifestations of IgAN include persistent asymptomatic microscopic haematuria or gross haematuria, accompanied by varying degrees of albuminuria and hypertension; in severe cases, renal failure may occur [6]. IgAN stands as a leading cause of chronic kidney failure, with approximately 40% of patients progressing to end-stage renal disease (ESRD) within 10 to 20 years, imposing a substantial economic burden on society and families [4, 7]. Currently, there is no standardized treatment for IgAN; supportive measures such as protein control, blood pressure regulation, and sodium intake management constitute the mainstay of therapy [3, 8, 9]. In clinical practice, angiotensin-converting enzyme inhibitors or angiotensin receptor blockers are commonly employed to manage albuminuria and blood pressure levels; Some individuals receive corticosteroids or combined immunosuppressants. However, conflicting results have been reported from studies investigating these interventions [3, 10].

IgAN is an autoimmune disease, and numerous studies have demonstrated that the pathogenesis of IgA involves the formation and deposition of immune complexes consisting of galactose-deficient IgA1 (Gd-IgA1) and autoantibodies against Gd-IgA1 (anti-Gd-IgA1 antibodies) in the glomeruli, along with activation of the complement system [5, 11–15]. It is widely accepted that following infection, patients produce significant amounts of Gd-IgA1 in the hinge region, which is characterized by galactose-deficient O-glycan. Subsequently, they generate IgG autoantibodies targeting terminal N-acetylgalactosamine (GalNAc) residues, leading to the formation of IgG-Gd-IgA1 immune complexes primarily deposited in the mesangial region of glomeruli. This triggers complement molecule activation and initiates inflammatory responses, ultimately resulting in disease development [16–20]. Biologic agents targeting immune pathogenesis offer a novel therapeutic approach for controlling IgAN progression by depleting or regulating B cells, plasma cells, alternate or lectin pathways involved in Gd-IgA1 production [21–26]. Therefore, this study aims to evaluate both the efficacy and safety aspects of biologic agents for the treatment of IgAN while providing evidence-based references.

## Materials and methods

### Study registration

Our study protocol is designed according to the Preferred Reporting Items for Systematic Review and Meta-Analysis Protocols (PRISMA-P) reporting checklist (S1 Checklist) [27, 28]. The protocol for this study has been registered in the International Prospective Register of Systematic Reviews (PROSPERO): CRD42023400450. Ethical approval is not required for this study.

### Inclusion criteria

The inclusion criteria will be framed based on the PICOS format [29, 30].

#### Study participants (P)

Adult and pediatric patients with biopsy-proven IgA nephropathy, regardless of gender, age, ethnicity, race, or course of disease.

#### Interventions (I)

Biologic agents, including rituximab, sibeprenlimab, velcade, narsoplimab, iptacopan, felzartamab, telitacicept, tarpeyo, atacicept, avacopan, and BION-1301.

#### Controls (C)

Placebo.

#### Outcomes (O)

The primary outcome is remission rate, while secondary outcomes include 24-hour urine protein excretion, serum albumin, blood pressure, serum creatinine (Scr), and estimated glomerular filtration rate (eGFR). The safety index refers to the incidence of adverse reactions (AE).

#### Study design (S)

Randomized controlled trial (RCT).

### Exclusion criteria

Exclusion criteria include case reports, observational studies, reviews, meta-analyses, animal studies, or trials with an active drug as comparator.

### Search strategy

We will conduct a systematic search for relevant RCTs in PubMed, EMbase, Web of Science, Cochrane Library, and www.clinicaltrials.gov from the inception of each database to October 2023. MeSH terms and free words will be utilized for the search strategy. The detailed search strategy for PubMed is provided in Table 1.

**Table 1:** Search strategy for PubMed.

Search	Query
#1	Search: ((((((IgA Nephropathy[MeSH Terms]) OR (IgA Nephropathy[Title/Abstract])) OR (immunoglobulin A nephropathy[Title/Abstract])) OR (IgAN[Title/Abstract])) OR (IgA nephritis[Title/Abstract])) OR (IgA glomerulonephritis[Title/Abstract])) OR (Berger’s disease[Title/Abstract])
#2	Search: ((((Biologic Agents[MeSH Terms]) OR (Biologic Agents[Title/Abstract])) OR (Biological Agents[Title/Abstract])) OR (Biological Factors[Title/Abstract])) OR (Biologic Factors[Title/Abstract])
#3	Search: ((((((((((rituximab[Title/Abstract]) OR (sibeprenlimab[Title/Abstract])) OR (velcade[Title/Abstract])) OR (narsoplimab[Title/Abstract])) OR (iptacopan[Title/Abstract])) OR (felzartamab[Title/Abstract])) OR (telitacicept[Title/Abstract])) OR (tarpeyo[Title/Abstract])) OR (atacicept[Title/Abstract])) OR (avacopan[Title/Abstract])) OR (BION-1301[Title/Abstract])
#4	Search: #2 OR #3
#5	Search: (((randomized controlled trial[Publication Type]) OR (controlled clinical trial[Publication Type])) OR (randomized[Title/Abstract])) OR (randomly[Title/Abstract])
#6	Search: #1 AND #4 AND #5

### Study selection and data extraction

Two independent researchers (JM and YPZ) will search the literature separately, and the results will be imported into EndNote X9 (Clarivate). After removing duplicates, the literature that meets the conditions will be screened by reading the titles and abstracts according to the confirmed inclusion and exclusion criteria. Microsoft Excel will be used to extract the first author, publication year, country, sample size, sex, average age, details of the intervention and control regimen (i.e., types of biologic agent, dosage, route of administration, duration), outcome measures (i.e., remission rate, 24-hour urine protein excretion, serum albumin, blood pressure, serum creatinine, and estimated glomerular filtration rate, and the incidence of adverse events), follow-up time points of the outcome, and methods of data analysis (intention-to-treat population or per-protocol analysis).

**Fig 1:**
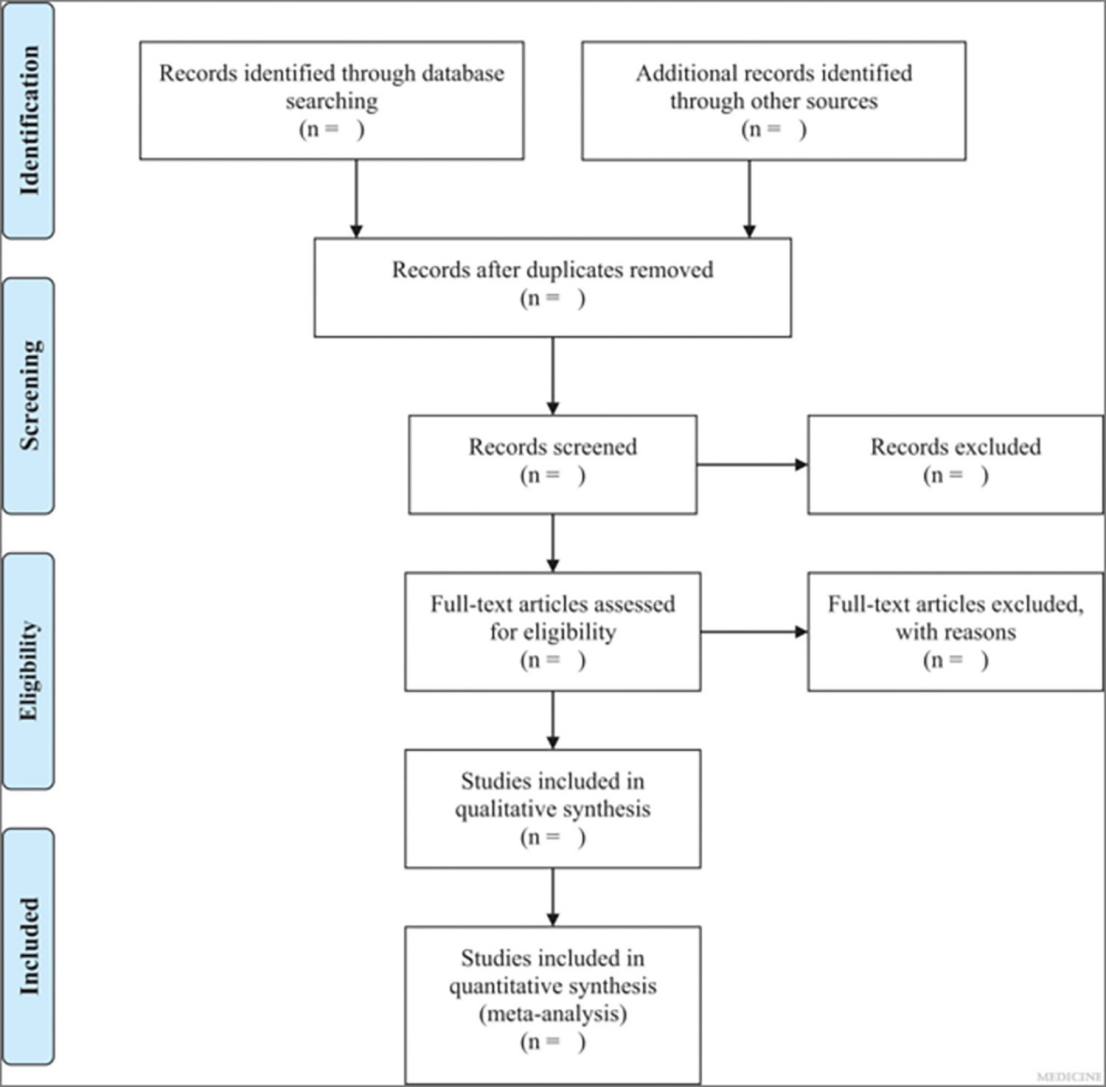
PRISMA-2020 flow diagram.

The remission rate is defined as the sum of complete and partial remissions [31–33]. Complete remission (CR) is characterized by a 24-hour urinary protein level below 0.3 g and stable serum creatinine (with fluctuations less than 15%). Partial remission (PR) is defined as a 24-hour urinary protein level between 0.3 g and 3.5 g, with more than a 50% reduction from baseline, and stable serum creatinine levels. Cases that do not meet these criteria are considered ineffective in achieving remission. Additionally, we will make it clear whether the number of observations in the study should match the number of "units" that are randomized. In case of missing data, efforts will be made to contact the corresponding author for retrieval. If it is not possible to obtain relevant data, only the available data will be analyzed and discussed as a limitation of the study. Any inconsistencies in the information will be resolved by a third investigator (JYX). The PRISMA-2020 flow diagram for this study is shown in Fig 1.

### Risk of bias assessment

Two independent researchers (JM and YPZ) will evaluate the risk of bias using version 2 of the Cochrane tool for assessing the risk of bias in randomized trials (RoB 2) [34]. The tool is formed by five domains with different questions: (1) Bias arising from the randomization process; (2) Bias due to deviations from intended interventions; (3) Bias due to missing outcome data; (4) Bias in measurement of the outcome; (5) Bias in selection of the reported result. A study will be considered to be at a “low risk of bias” if all five domains have been judged to be at a low risk of bias. A study will be considered to have “some concerns” if it has been judged to raise some concerns in at least one domain. A study will be considered to be at a “high risk of bias” overall if it is judged to be at a high risk of bias in at least one domain [34–36]. In case there are inconsistencies, the third investigator (JYX) will make the final determination.

### Statistical analysis

The meta-analysis will be conducted using Revman 5.4.1 software (Cochrane Collaboration). Dichotomous variables will be analyzed using risk ratio (RR) with a 95% confidence interval (CI), while continuous variables will be analyzed using mean difference (MD) or standardized mean difference (SMD) with a 95% CI.

#### Assessment of heterogeneity

Statistical heterogeneity will be assessed through the Cochran Q test and *I*^*2*^ index, which measure the percentage of total variation across studies that is due to heterogeneity rather than chance [37]. *I*^*2*^ values lie between 0 percent and 100 percent, with more than 50 percent considered to have substantial heterogeneity. For the pooling of estimates across the included studies, the summary RRs and their 95% CIs will be calculated using a random effects model, based on the presence of statistical heterogeneity in the studies. Otherwise, a fixed effects model will be used [38, 39].

#### Subgroup analysis and sensitivity analysis

In the presence of significant heterogeneity, subgroup analysis should be conducted based on study, participant, and outcome characteristics, as well as methodological factors such as sex, race, age, different types of biologic agent, dosage, and duration of intervention. In the absence of clear sources of heterogeneity, we will provide a detailed descriptions of the variables used in the subgroup analysis and their results, ultimately finding no significant influence factors for the heterogeneity. Sensitivity analysis will be used to investigate the stability of the results by omitting one study at a time [40, 41].

#### Publication bias

To assess publication bias, funnel plots and Egger tests will be utilized [42].

### Quality of evidence

We will employ GRADEprofiler 3.6 (Cochrane Collaboration) to evaluate the quality of the evidence based on the risk of bias, indirectness, inconsistency, imprecision, and publication bias [43]. The quality of the evidence will be categorized as very low certainty, low certainty, moderate certainty, or high certainty [44, 45]. High certainty: We are very confident that the true effect is close to the effect estimate. Moderate certainty: We have moderate confidence in the effect estimates: the true effect may be close to the effect estimates, but it is also possible that it may be significantly different. Low certainty: We have limited confidence in the effect estimates: the true effect may be quite different from the effect estimates. Very low certainty: We have little confidence in the effect estimates: The true effect may be quite different from the effect estimates. Evidence should be classified as "no limitation or not serious" (not important enough to warrant downgrade) if no reason is found for it to be downgraded in one of the five domains of quality assessment. If a cause of evidence downgrade is found, it should be classified as "serious" (downgrading the certainty rating by one level) or "very serious" (downgrading the certainty grade by two levels) [30].

## Discussion

IgAN was initially proposed by Jean Berger in 1968 and is currently recognized as the most prevalent primary glomerulonephritis worldwide [46]. Despite substantial advancements in comprehending its pathophysiology over the past five decades, there remains a dearth of efficacious clinical treatment options [47–49]. None of the therapeutic regimens effectively treats or prevents IgAN progression [50]. Consequently, there exists an unmet medical need for more efficacious and safer therapies for IgAN patients who are at high risk of progressive kidney disease. As research continues to unravel the immunopathology underlying this condition, biologic agents represent a novel therapeutic approach that can impede or halt disease progression by targeting its core pathogenesis [25]. The results may help guide future guidelines for the treatment and management of IgAN.

## Supporting information

S1 Checklist PRISMA-P (Preferred Reporting Items for Systematic Review and Meta-Analysis Protocols) 2015 checklist: Recommended items to address in a systematic review protocol. (DOC)

## Abbreviations

IgAN: IgA nephropathy
IgA: immunoglobulin A
ESRD: end-stage renal disease
Gd-IgA1: galactose-deficient IgA1
RCTs: randomized controlled trials
Scr: serum creatinine
eGFR: estimated glomerular filtration rate
AE: adverse reactions
